# Extracorporeal photopheresis induces the release of anti-inflammatory fatty acids and oxylipins and suppresses pro-inflammatory sphingosine-1-phosphate

**DOI:** 10.1007/s00011-025-02007-6

**Published:** 2025-02-13

**Authors:** Gerhard Hagn, Ara Cho, Nina Zila, Barbara Sterniczky, Christian Jantschitsch, Dexin Dong, Andrea Bileck, Mariia Koren, Philipp Paulitschke, Thomas Mohr, Robert Knobler, Wolfgang Peter Weninger, Christopher Gerner, Verena Paulitschke

**Affiliations:** 1 Department of Analytical Chemistry, University of Vienna, Währinger Street 38, 1090 Vienna, Austria; 2 Department of Dermatology, Medical University of Vienna, Währinger Gürtel 18-20, 1090 Vienna, Austria; 3 Department Health Sciences, Section Biomedical Science, University of Applied Sciences FH Campus Wien, Vienna, Austria; 4 Joint Metabolome Facility, University and Medical University of Vienna, Vienna, Austria; 5 PHIO scientific GmbH, 81371 Munich, Germany

**Keywords:** Photopheresis, ECP, UVA, Lipidomics, Proteomics, Molecules sphingosine-1-phosphate, PPPM, Clinically relevant lipid profiles, Individual lipidomics, New therapeutic concepts, Inflammation

## Abstract

**Aims:**

Extracorporeal photopheresis (ECP) is a UVA-based phototherapy of whole blood and well established as a first line or combination therapy for the treatment of cutaneous T-cell lymphoma, systemic sclerosis, graft-versus-host disease and is used to control organ transplant rejection. While the proapoptotic activity on activated T-cells is evident, the clinical efficacy of this treatment also appears to be based on other yet unknown mechanisms. In this study, we aimed to identify novel mechanisms of ECP regardless of the patient’s background situation.

**Main methods:**

To better understand the immediate consequences of ECP, we analyzed blood plasma of patients with different ECP indications immediately before and after treatment with regard to proteins and lipid mediators.

**Key findings:**

While proteome profiling identified substantial inter-individual differences in the protein composition, no significant alteration was detectable upon treatment. In contrast, several fatty acids and lipid mediators were found to be significantly altered by ECP. Remarkably, upregulated lipid mediators including polyunsaturated fatty acids, 12-HEPE and 13-OxoODE have been described to be anti-inflammatory, while the downregulated molecules sphingosine-1-phosphate (S1P) and stearic acid are potent pro-inflammatory mediators. A selective sphingosine-1-phosphate-1 receptor (S1P1) modulator AUY954, which decreases S1P1 and experimentally reduces transplant rejection in vivo, showed greater anti-proliferative activity in human lung fibroblasts from COPD patients compared to normal lung fibroblasts, confirming that this pathway may be important in ECP and its mode of action.

**Significance and outlook:**

In conclusion, we suggest that the ECP-induced changes in lipid mediators may contribute to the remarkable anti-inflammatory effects of the treatment. Depending on their lipid status, patients may benefit from novel treatment regimens combining ECP with lipid modulators. This could be used for the prevention of transplant organ rejection, the treatment of acute or chronic GvHD or transplant organ rejection and the long-term treatment of various skin diseases. This study uncovers novel mechanisms of ECP, that can be used to establish clinically relevant lipid profiles of patients to support patient stratification, predictive or prognostic purposes and thus personalized medical care in the framework of PPPM practice. A combination with S1P modulators may therefore have beneficial effects.

**Supplementary Information:**

The online version contains supplementary material available at 10.1007/s00011-025-02007-6.

## Introduction

Extracorporeal photopheresis (ECP) is a well-tolerated treatment option for multiple clinical indications. This leukapheresis-based therapy derived from the field of a dermatological treatment modality, the so called PUVA treatment - psoralen plus Ultraviolet A (UVA) - and was initially introduced for treatment of steroid-refractory patients with cutaneous T-cell lymphoma (CTCL) [1]. Key features of using phototherapy including PUVA, UVB and UVA1 are proapoptotic, immunomodulatory, antipruritic, antifibrotic, pro-pigmentary, and pro-prebiotic, consequently inducing improvement of the skin manifestation [2]. Molecular profiling methods such as proteomics and lipidomics can help to better understand which molecules can mediate the observed effects.

Due to its excellent safety profile, especially its beneficial effect of reducing the need for systemic steroids, ECP found continuous expansion of indications and is nowadays used for dermatologic diseases, acute and chronic graft-versus-host disease (GvHD), treatment of acute and chronic solid organ transplantation, as well as for rare autoimmune diseases [3–6]. In addition, we and others are currently evaluating ECP as a tool to support the prevention of transplant rejection [7]. A better understanding of the mode of action may substantially improve the present treatment indications, supporting preventive medicine, and may help to design synergistic combinations with other strategies supporting personalized medicine.

*Via* peripheral access from a cubital vein of a patient whole blood is collected followed by centrifugation and isolation of a leukocyte whole cell suspension, the so-called buffy coat. In the next step, the photo-activator 8-methoxypsoralen (8-MOP) is added to the cell solution, irradiated with UVA light and reinfused to the patient with an anticoagulation solution, usually acid citrate dextrose (ACD) or heparin. ECP treatments are carried out on two consecutive days and usually start every two weeks for a certain period of time.

Over the past 35 years, great efforts have been made to find the reason for the clinically proven efficacy, including the intention to explore new indications for ECP treatments and to find biomarkers to understand and identify the patients who will benefit most from the treatment [8–10].

Results of early studies indicate that the therapeutic effect of ECP is based on the initiation of apoptosis in lymphoid cells through the combination of the photosensitizer 8-MOP and UVA light. UVA irradiation of cells after exposure to 8-MOP was shown to induce DNA cross-linking [11–13].

Other trials observed alterations in the cytokine profile of peripheral blood, for example increase of tumor necrosis factor alpha (TNF-α) and interleukin (IL)-6 [14], while further studies have demonstrated the differentiation of T-cells into different cell subpopulations, in particular regulatory T-cells [15, 16]. Regulatory T-cells (Tregs) have already been extensively studied and are known to play a key role in increasing immunotolerance and hence preservation of transplanted organs and improvement of survival rates [17, 18].

Although a complete understanding and full elaboration of its mode of action is still required, several papers have demonstrated empirical evidence of the clinical benefit of ECP in various diseases and indications. In CTCL, multiple clinical studies showed beneficial effects up to complete remission; therefore, ECP is used as a first line treatment in CTCL with blood involvement, also known as Sézary Syndrome (SS) [19–21]. Recently published guidelines provide further recommendations to initiate ECP as second line or rescue treatment in therapy-refractory forms of mycosis fungoides (MF), a unique subunit of CTCL [5].

ECP is implemented in the current guidelines for the treatment of GvHD as second line therapy option in steroid-refractory presentations of GvHD [22, 23].

Over the last decades, extensive evidence has been accumulated for use of ECP in the context of solid organ transplantation, especially in heart and lung transplantation for the treatment of acute and chronic organ rejection [7, 24, 25]. ECP treatments have been as well analyzed as a prophylactic therapy option [26]. Recently, transcriptomics data from tissue biopsies in patients treated with ECP for chronic rejection after renal transplantation showed a reduction in fibrotic and inflammatory transcriptomic profiles [27].

To date, there are no molecular profiling data for plasma from ECP-treated patients. We used different indications for ECP, acute and chronic rejection in heart transplants and chronic GvHD, as well as various skin diseases such as systemic sclerosis (a chronic autoimmune disease) and cutaneous T-cell lymphoma. In addition, ECP is evaluated in a prophylactic setting in transplant patients. Therefore, ECP is used or evaluated at the primary, secondary and tertiary level of prevention. In our setting, we analyzed patients at the most frequently used level, tertiary prevention, in various ECP indications.

The aim of this study is to determine changes in protein and/or oxylipin levels induced by ECP treatment in order to gain more insight into the possible mechanisms of action, using mass spectrometry-based analysis methods. A better understanding of the mode of action of ECP will help to use this therapeutic option in a more systematic fashion, will support further individual optimization of therapeutic concepts and thus contribute to a rational patient stratification in PPPM practice.

## Materials and methods

### Sample acquisition

We performed a prospective, explorative pilot study, in which we recruited 6 patients who had not previously received ECP treatments, regardless of the indication for ECP. 24 plasma samples were collected before and after ECP procedure on both treatment days. ACD was used as anticoagulant during the collection of 1500 mL of whole blood. The whole procedure is performed as previously described [28].

Plasma samples were collected following a strict protocol starting with drawing blood with standard ethylenediaminetetraacetic acid (EDTA) Vacuette tubes followed by centrifugation for 15 min at room temperature with 720 g. Plasma was then collected and stored in 500 µl aliquots in clear flat glass vials with screw caps to be stored at -80 °C. Exact time of blood drawing, starting time of centrifugation as well as freezing time were documented.

The work described has been carried out in accordance with The Code.

of Ethics of the World Medical Association (Declaration of Helsinki) for.

experiments involving humans. All procedures were performed in compliance with relevant laws and institutional guidelines and have been approved by the appropriate institutional committee (EK No.: 2076/2015).

### Sample preparation for oxylipin and fatty acids analyses

The analysis of oxylipins and fatty acids was performed as previously described [29]. Plasma (500 µL) was thawed on ice and added to a 15 mL Falcon™ tube containing ice cold ethanol (2 mL; EtOH) and internal standards (12 S-HETE-d8, 15 S-HETE-d8, 20-HETE-d6, 5-oxo-eicosatetraenoic acid (OxoETE)-d7, prostaglandin E2 (PGE2)-d4 and 11,12-dihydroxy-eicosatrienoic acid (DiHETrE)-d11 (Cayman Chemical, Tallinn, Estonia)). Exact concentrations of the internal standards can be found in Supplementary Table S3. After vortex mixing, the samples were stored at -20 °C overnight resulting in suspension of proteins. The samples were then centrifuged (30 min, 4536 g, 4 °C), the supernatant was transferred into a new 15 mL Falcon™ tube, and the original sample volume (500 µL) was restored *via* evaporation of EtOH at 37 °C by vacuum centrifugation. Afterwards, the samples were loaded on preconditioned StrataX solid phase extraction (SPE) columns (30 mg mL^− 1^, Phenomenex, Torrance, CA, USA) using Pasteur pipettes. The SPE columns were washed with ice-cold MS grade water (5 mL; VWR International, Vienna, Austria) and analytes were eluted with ice-cold MS grade methanol (MeOH) (500 µL; VWR International, Vienna, Austria) including 2% formic acid (FA) (≥ 99%; VWR International, Vienna, Austria). Samples were dried using a gentle nitrogen stream at room temperature, reconstituted with 150 µL reconstitution buffer (H_2_O/acetonitrile (ACN)/MeOH + 0.2% FA– vol% 65:31.5:3.5) and subsequently measured *via* LC-MS/MS.

### LC–MS/MS analysis

For the LC-MS analyses, a Vanquish™ ultra-high-performance LC (UHPLC) system (Thermo Fisher Scientific™, Vienna, Austria) was coupled to a high-resolution quadrupole orbitrap mass spectrometer (Thermo Fisher Scientific™ QExactive™ HF hybrid quadrupole orbitrap mass spectrometer). For separation of analytes the Vanquish™ UHPLC system was equipped with a reversed-phase Kinetex^®^ C18 column (2.6 μm XB-C18, 100 Å, LC column 150 × 2.1 mm, Torrance, CA, USA). The injection volume was 20 µL, the flow rate was set to 200 µL min^− 1^, the LC column oven was set to 40 °C and the autosampler was set to 4 °C. All samples were measured in technical duplicates. The total run time was 20 min with a gradient flow profile starting at 35% B and increasing to 90% B (1–10 min). After further increasing to 99% B within 0.5 min and keeping it for 5 min, solvent B was decreased to 35% B within 0.5 min and held for 4 min to equilibrate the column. Mobile phase A was H_2_O + 0.2% FA and mobile phase B was ACN: MeOH (vol% 90:10) + 0.2% FA. The QExactive™ HF hybrid quadrupole orbitrap mass spectrometer was equipped with a HESI source operating in negative ionization mode with a spray voltage of 3.5 kV. The capillary temperature was set to 253 °C, sheath gas and auxiliary gas were set to 46 and 10 arbitrary units, respectively. The scan range on the MS1 level was 250–700 m/z with a resolution of 60,000 (*m/z* 200). On the MS2 level the resolution was 15,000 (*m/z* 200). Here, a top 2 data-dependent acquisition (DDA) method was applied using an HCD collision cell with a normalized collision energy of 24. Therefore, 33 m/z values from an inclusion list, which are specific for oxylipins and their precursor fatty acids, were preferentially selected for fragmentation (Supplementary Table S4).

### LC–MS/MS Data Processing

For the data analysis, raw files generated by the QExactive™ HF mass spectrometer were checked manually using the Xcalibur™ Qual Browser software (version 4.1.31.9; Thermo Fisher Scientific™, Vienna, Austria) by comparing reference spectra from the LIPIDMAPS depository library from July 2020 [30]. The identification of analytes was performed based on exact mass, retention time and MS/MS fragmentation pattern (Supplementary Table S5). Due to the high structural variety of oxylipins, isomers of known oxylipin species which were not yet identified *via* commercially available standards have been designated as “molecular mass_chromatographic retention time”. In addition, trans-fatty acid isoforms of polyunsaturated fatty acids such as DPA were designated as “isoform I”, “isoform II” or else. For relative quantification, the TraceFinder software (version 4.1; Thermo Fisher Scientific™, Vienna, Austria) was applied, allowing a mass deviation of 5 ppm. The resulting data containing the peak areas of each analyte were exported and read using the R software package (version 4.2.0) [31]. The peak areas were log_2_-transformed and normalized to the internal standards. Therefore, the log_2_-transformed mean peak area of the internal standards was subtracted from the log_2_-transformed analyte areas to correct for variances arising from sample extraction and LC-MS/MS analysis. To obtain values similar to proteomics LFQ values and thus, enable imputation of missing values, 20 was added to the log_2_-transformed areas. For the imputation of missing values, the minProb function of the imputeLCMD package (version 2.1) [32] was used. The log_2_-transformed normalized peak areas of oxylipins and fatty acids are designated here (Supplementary Table S6). For statistical analysis, the log_2_-transformed normalized peak areas were fitted to a linear model using the LIMMA package and samples before and after ECP treatment were pairwise compared using the empirical Bayes method [33]. Resulting p-values < 0.05 were considered as statistically significant (Supplementary Table S6). For the multiple testing correction, the Benjamini-Hochberg procedure was applied to all p-values. Volcano plots were produced using the ggrepel (version 0.9.1) and mdthemes packages (version 0.1.0) [34, 35].

### Plasma proteomics

#### Sample Preparation for digestion of proteins

Briefly, frozen EDTA-anticoagulated plasma samples were thawed on ice, diluted 1:20 in lysis buffer (8 M urea, 50 mM triethylammonium bicarbonate (TEAB), 5% sodium dodecyl sulfate (SDS)) and heated at 95 °C for 5 min. Afterwards, the protein concentration was determined using a BCA assay and 20 µg of protein was used for the digestion of proteins according to the ProtiFi S-Trap™ protocol [36]. Here, disulfide bonds of the solubilized proteins were reduced using 64 mM dithiothreitol (DTT) and protected *via* carbamidomethylation using 48 mM iodoacetamide (IAA). Upon adding trapping buffer (90% v/v MeOH, 0.1 M TEAB), samples are loaded onto S-trap mini cartridges, washed, and digested using a Trypsin/Lys-C Mix (1:40 enzyme to substrate ratio) at 37 °C for two hours. The resulting peptides were eluted, dried, and stored at minus 20 °C until LC-MS/MS analysis.

#### Untargeted LC-MS/MS analysis of proteins

For the untargeted proteomics approach, dried peptide samples were reconstituted using 5 µL of 30% FA containing 4 synthetic standard peptides at a concentration of 10 fmol and diluted with 40 µL of loading solvent (97.95% H_2_O, 2% ACN, 0.05% trifluoroacetic acid (TFA)) as previously described [37, 38]. The chromatographic separation was performed using a Dionex Ultimate™ 3000 nano UHPLC system (Thermo Fisher Scientific™), equipped with a pre-column (2 cm × 100 μm, 5 μm, 100 Å, C18 Acclaim Pep-Map™ 100; Thermo Fisher Scientific™) for pre-concentration and an analytical column (25 cm × 75 μm, 1.6 μm, 120 Å, C18, Aurora Series emitter column; IonOpticks). The injection volume was 1 µL, and the flow rate for the pre-concentration was 10 µL/min using mobile phase A (99.9% H_2_O, 0.1% FA). For the separation of peptides, a flow rate of 300 nL/min was applied with a gradient flow profile starting at 7% mobile phase B (79.9% ACN, 20% H_2_O, 0.1% FA), which is increased to 40% B over 43 min, resulting in a total run time of 85 min including washing an equilibration of the column. For the mass spectrometric analysis, the Dionex Ultimate™ 3000 nano UHPLC system was coupled with the timsTOF Pro mass spectrometer (Bruker) equipped with a captive spray ion source operating at 1650 V. The MS was operating in Parallel Accumulation-Serial Fragmentation (PASEF) mode with a moderate MS data reduction applied. The MS scan range was 100 to 1700 *m/z* on the MS1 and MS2 level, the 1/k0 scan range was 0.60 to 1.60 V*s/cm^2^, resulting in a ramp time of 100 ms for trapped ion mobility separation. Ten PASEF MS/MS scans per cycle were leading to a total cycle time of 1.16 s. In addition, the collision energy was ramped from 20 to 59 eV as a function of enhancing ion mobility, and the quadrupole isolation width was 2 Th for *m/z* < 700 and 3 Th for *m/z* > 700.

#### Data analysis of proteins

Raw files generated by the timsTOF Pro mass spectrometer were analyzed using the MaxQuant 1.6.17.0 software package running the Andromeda search engine [39]. For protein identification and LFQ, raw data were searched against the SwissProt database ‘‘homo sapiens’’ (version 141219 with 20380 entries) with a tolerance of 20 ppm at the peptide level and maximal two missed cleavages. Carbamidomethylation on cysteines was set as a fixed modification, while N-terminal protein acetylation and methionine oxidation were set as variable modifications. A minimum of one unique peptide was required for positive identification, and the ‘‘match between runs’’ option was applied with a match window of 0.7 min, an alignment time window of 20 min, a match ion mobility window of 0.05 and an alignment ion mobility of 1. For peptide and protein identifications, an FDR ≤ 0.01 was set, which was computed based on a reversed decoy database. Subsequent data processing and evaluation were performed using the Perseus software (version 1.6.14.0) with reversed sequence and common contaminant filtering [40]. The sample annotation was performed according to the respective study cohorts, LFQ intensity values were log_2_-transformed, and proteins were filtered for a minimum number of 5 independent identifications in at least one group. Missing values were imputed from normal distribution with a width of 0.3 and a down shift of 1.8. The statistical analysis and data visualization were performed using the Perseus software (version 1.6.14.0).

#### Fragpipe pipeline and PSVA

Mass spectrometry data were searched and quantified using the fragpipe pipeline [41, 42] with the Uniprot-Swissprot curated proteins database [43]. The resulting LFQvalue matrix was log2 transformed, quantile normalized, and MNAR values were imputed using the MinProb imputation from the R package DEP [44]. Differentially expressed proteins were determined using the LIMMA package [33] with timepoint as variable, and patientID as a blocking factor. P-values where adjusted according to Benjamini Hochberg [45]. Protein Set Variation Analysis (PSVA) was done using the package GSVA [46] from R with the molecular signature database [47] as an input. Sets from the molecular signature database were filtered to biologically relevant pathways using Gene Ontology (GO) terms and Reactome. Differentially regulated pathways were determined by applying a linear model as described above. The proteins of the most regulated pathway on day 1 and day 2 were inserted into the String database (STRING: functional protein association networks, Version 12.0).

#### Viability assay

Normal human lung fibroblasts (NHLF, Lonza #CC-2512) and diseased human lung fibroblasts from COPD patients (DHLF-COPD, Lonza #00195277) were seeded in 96-well plates at a density of 2.5 × 10^3^ cells per well. After 24 h, the cells were treated with the sphingosine-1-phosphate-1 receptor (S1P1) agonist N-[[2-[2-(trifluoromethyl)[1,1’-biphenyl]-4-yl]benzo[b]thien-5-yl]methyl]-β-alanine (Cayman Chemical AUY954, #9000548) at various concentrations and incubated for 72 h. Fibroblast proliferation and viability were determined using a standard colorimetric cell proliferation assay (Promega CellTiter 96^®^ AQueous One Solution Cell Proliferation Assay (MTS), #G3580) at 490 nm. IC50 values were calculated by non-linear regression using the dose-response equations included in GraphPad Prism software version 10.3.1 (GraphPad Software Inc., USA).

#### Life cell imaging with PHIO cellwatcher M

Normal human lung fibroblasts (NHLF, Lonza #CC-2512) and diseased human lung fibroblasts from COPD patients (DHLF-COPD, Lonza #00195277) were seeded in 6-well plates at a density of 5 × 10^5^ cells per well. After 24 h, cells were treated with the sphingosine-1-phosphate-1 (S1P1) receptor agonist N-[[2-[2-(trifluoromethyl)[1,1’-biphenyl]-4-yl]benzo[b]thien-5-yl]methyl]-β-alanine (Cayman Chemical AUY954, #9000548) at 7821 nM, the IC50 concentration of DHLF-COPD determined in the viability assay. Cell proliferation was monitored in real time in 6-well plates using a PHIO Cellwatcher M microscope (PHIO scientific GmbH, Munich, Germany) [48] placed inside the incubator, imaging each well every 30 min over a 70 h period. The proliferation assays were analyzed using the PHIOme Data Management and Analysis Platform software add-ons “Proliferation” version 1.4.2 (PHIO scientific GmbH, Munich, Germany). Two hypothesis tests were performed using the scipy package to evaluate differences between the cell types and groups Mann-Whitney U test: This non-parametric test assesses whether the distributions of two independent groups differ, focusing on overall ranks rather than specific metrics such as mean or median. The Mood’s Median test was also performed:

This is a non-parametric test specifically designed to compare medians between groups. It evaluates whether the central tendency (median) is significantly different between groups.

## Results

**Fig. 1:**
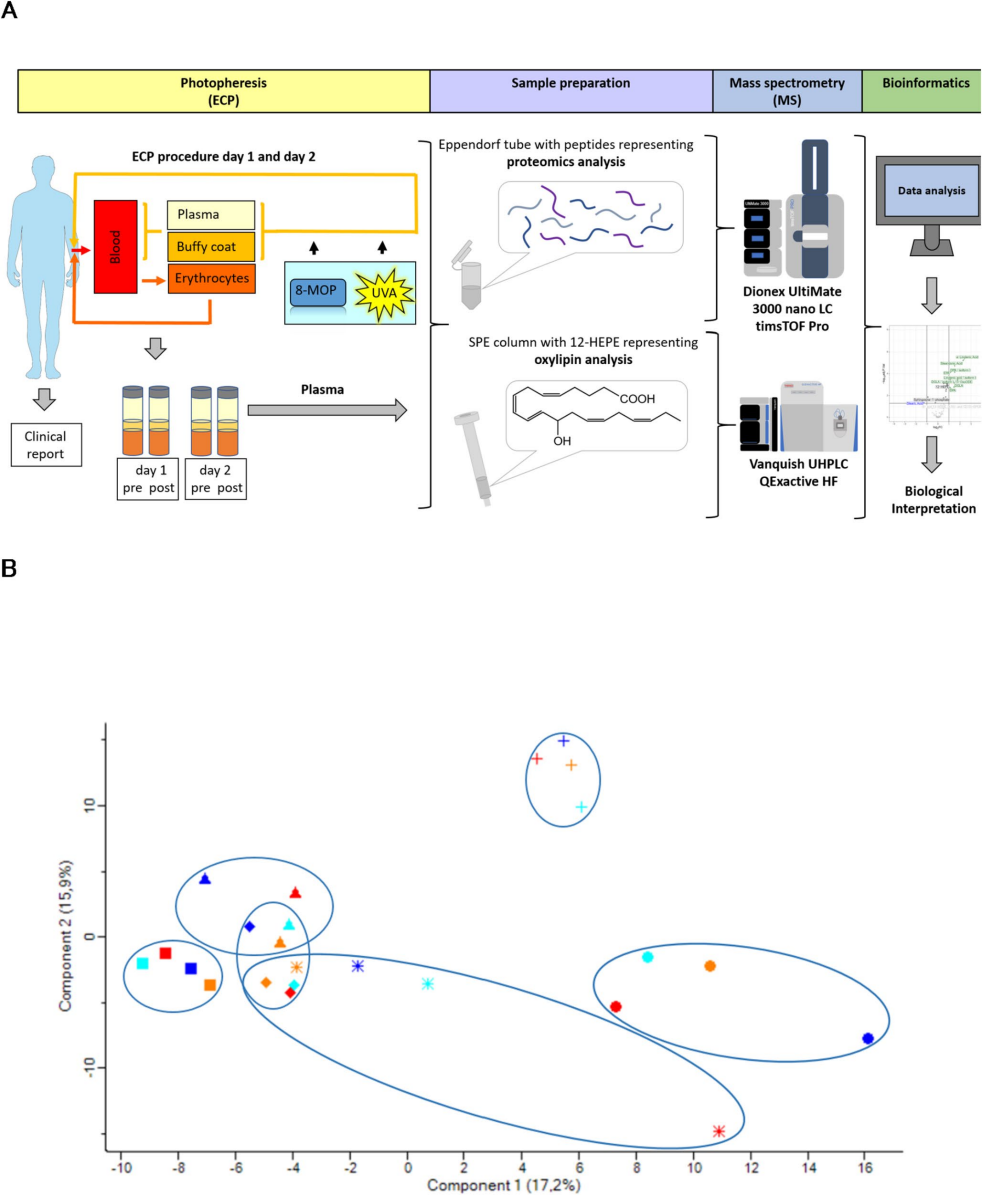
**A**: Conceptual figure of the study approach. **B**: Principal component analysis of plasma proteome profiles of patients before (day 1 light blue, day 2 orange) and after (day 1 dark blue, day 2 red) ECP treatment. While the individual patients (encircled, marked with different symbols) clustered, there was apparently no uniform effect of ECP treatment. *ECP does not induce significant proteome alterations in blood plasma*

We collected blood samples from 6 ECP patients with 4 different diagnoses or clinical indications (Supplementary Table S1) before and after ECP at two consecutive days (day 1 and the following day 2) to further isolate plasma. Thus, 24 plasma samples were processed as described previously to perform liquid chromatography-mass spectrometry (LC-MS)-based proteome profiling as well as oxylipin analysis (Fig. 1A).

269 proteins were consistently (independent identification per protein in at least 9 out of 12 samples per analysis group) and reliably (false discovery rate (FDR) < 0.01 at protein and peptide level, at least 2 peptides per protein) identified and quantified based on relative label free quantification (LFQ) values. No protein was found with significantly different LFQ values (FDR < 0.05) when comparing plasma samples after treatment to the corresponding controls using a paired t-test. A principal component analysis demonstrates that the inter-individual variation was larger than any variation potentially attributed to treatment (Fig. 1B, Supplementary Table S2). Instead, the HTX patients indicated by triangle, square and rhomb are closely located. However, by using the fragpipe pipeline and GSVA, we identified “cellular response to lipid” (*p*-value: 0.02) and “regulation of plasminogen activation” (*p*-value: 0.012) as the most significantly regulated pathways at GO and Reactome level, pointing out the importance of the lipid metabolism and the possible involvement of the platelets in this context. The proteins involved in these pathways are interconnected, as demonstrated by STRING analysis (Supplementary Fig. 1A and B).

**Fig. 2:**
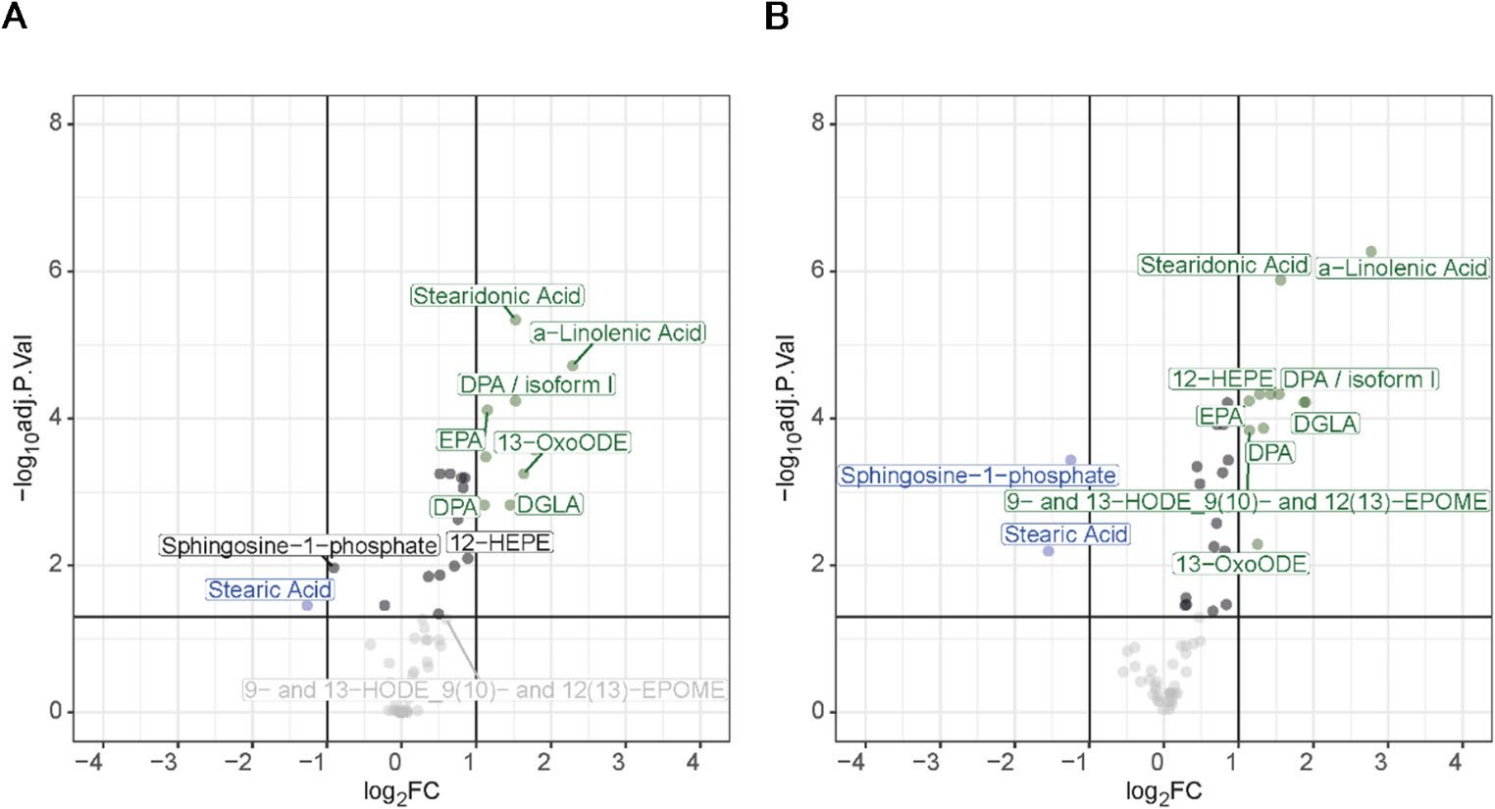
Volcano-Plots indicating up- and downregulated lipid mediators comparing **A**: day 1 after and before ECP; **B**: day 2 after and before ECP. For the statistical analysis, a paired t-test was performed and adjusted *p*-values according to the Benjamini-Hochberg procedure < 0.05 were considered as statistically significant. Log_2_FC is shown on the x-axis, -log_10_adj.Pval on the y-axis, downregulated lipid mediators in blue, unregulated in black and upregulated in green

### ECP induces significant alteration in the fatty acid and oxylipin composition of blood plasma

**Fig. 3:**
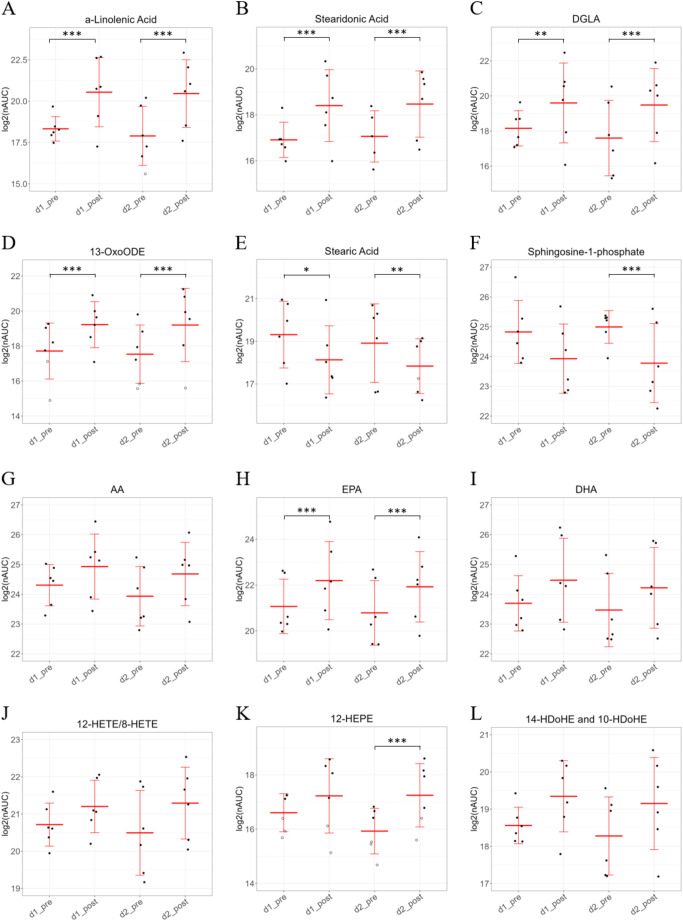
Box plots of indicated lipid mediators; **A**: α-Linolenic Acid; **B**: Stearidonic Acid; **C**: DGLA; **D**: 13-OxoODE; **E**: Stearic Acid; **F**: Sphingosine-1-phosphate; **G**: AA; **H**: EPA; **I**: DHA; **J**: 12-HETE/8-HETE; **K**: 12-HEPE and **L**: 14-HDoHE and 10-HDoHE. For the statistical analysis, a paired t-test was performed and adjusted *p*-values according to the Benjamini-Hochberg procedure. Adjusted *p*-values are indicated as follows: * 0.05, ** 0.01, *** 0.001

The analysis of fatty acids and oxylipins was performed as described previously [29]. After filtering for confidence (mass accuracy < 2ppm, matching isotopic pattern and fragmentation spectrum, retention time variation < 0.1%) and reproducible identifications (independent identification in at least 6 out of 12 samples per analysis group), a total of 67 lipids were identified and quantified according to normalized area under the curve values obtained by mass spectrometry. Normalization was performed with respect to internal standards as indicated in the Materials and Methods section. ECP treatment was found to induce several significant regulatory events (Figs. 2A, B and 3A–F, H and K).

**Fig. 4:**
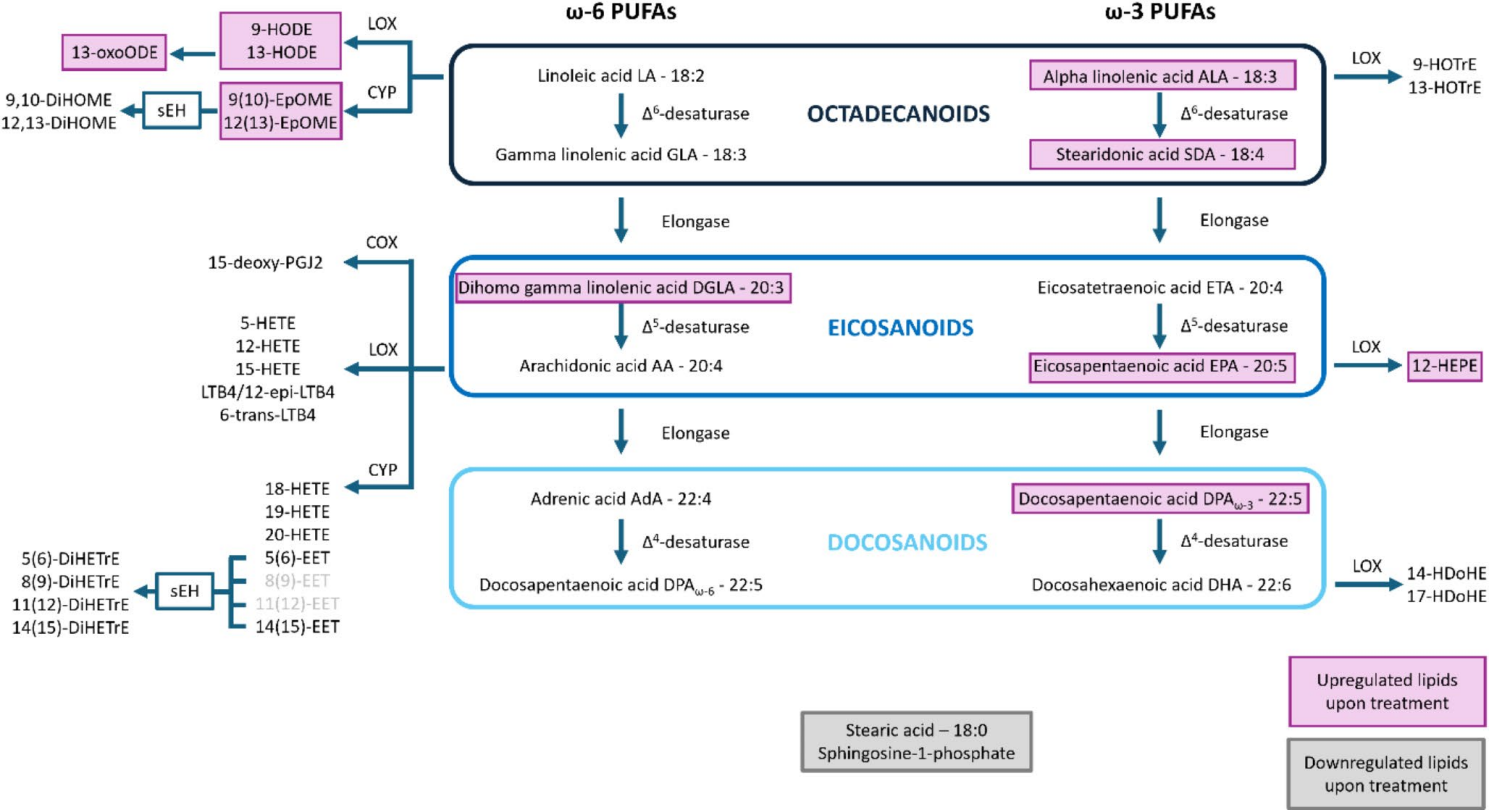
Overview of the omega-3 and omega-6 PUFA pathway and the oxylipins derived from it which were identified in the plasma samples before and after ECP treatment. Filled violet boxes represent significantly upregulated and filled grey boxes represent significantly downregulated compounds upon ECP treatment. The pathway was generated according to following citations: [49, 50]

Most striking was the upregulation of polyunsaturated fatty acids alpha-linolenic acid, stearidonic acid, eicosapentaenoic acid (EPA) and docosapentaenoic acid (DPA) together with trans-fatty acid isoforms (here designated as isoform I). This was accompanied by the downregulation of the unsaturated fatty acid stearic acid. Furthermore, several lipid mediators were found to be strongly affected by ECP treatment, including upregulation of 13-oxo-octadecadienoic acid (OxoODE), 12-hydroxy-eicosapentaenoic acid (HEPE) and downregulation of sphingosine-1-phosphate (Fig. 3D, K and F). These findings were highly similar at two independent treatment days and apparently independent of the patients’ background situation (Fig. 3). While the fatty acids arachidonic acid (AA) and docosahexaenoic acid (DHA) as well as the lipid mediators 12-hydroxy-eicosatetraenoic acid (HETE) and 14-hydroxy-docosahexaenoic acid (HDoHE) did not reach statistical significance, they were found to be upregulated upon treatment on both days 1 and 2 (Fig. 3G, I, J and L). Accordingly, comparison of the analysis results obtained before treatment at day 1 to the results obtained before treatment at day 2, as well as the analysis after treatment at day 1 to the corresponding results at day 2 did not reveal any significant alterations depicting the immediate regulation by ECP (Supplementary Fig. 1A, B). An overview of the oxylipins which were identified in the plasma of ECP treated patients can be found in Fig. 4.

**Fig. 5:**
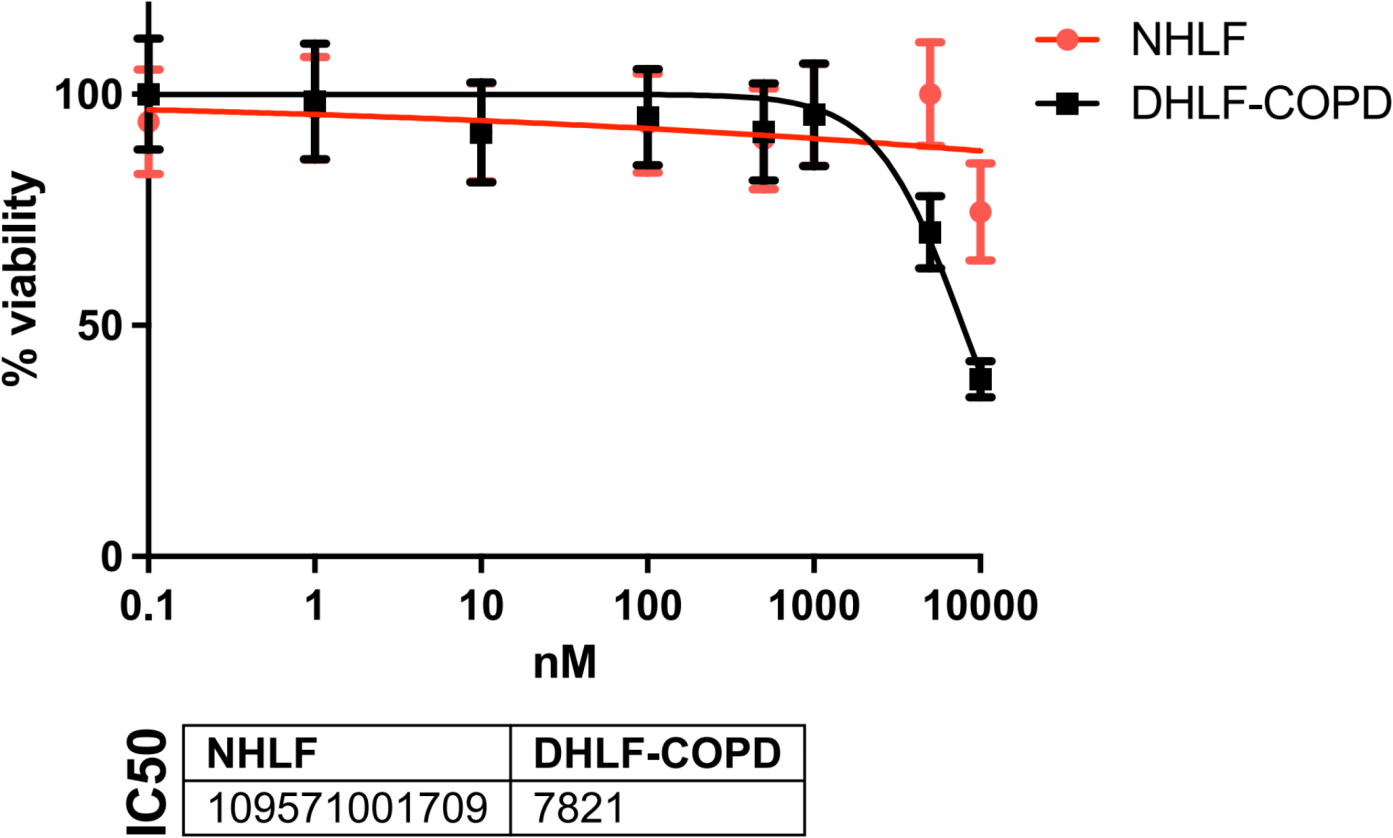
IC50 of NHLF and DHLF-COPD using AUY954: Normal human lung fibroblasts (NHLF) and diseased human lung fibroblasts from COPD patients (DHLF-COPD) were treated with the S1P receptor agonist AUY954 at various concentrations and incubated for 72 h. Proliferation and viability of fibroblasts were determined using a standard colorimetric cell proliferation assay. IC50 values were calculated by nonlinear regression using the dose–response equations integrated in GraphPad Prism software version 10.3.1. Four independent assays are pooled

**Fig. 6:**
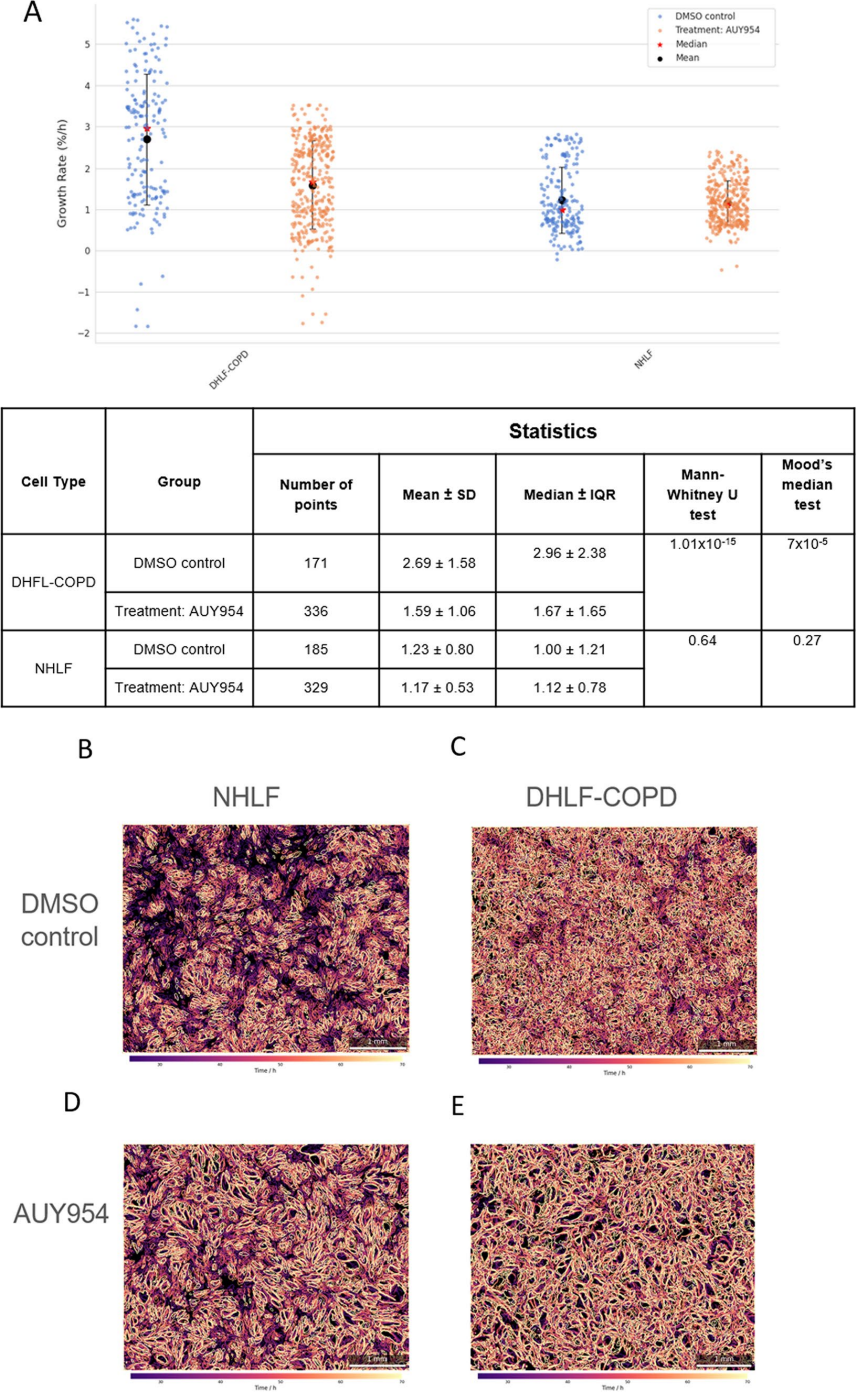
**A**: NHLF and DHLF-COPD were treated with AUY954 at 7821 nM or DMSO for 46 h. Cell proliferation was monitored in real time in 6-well plates using a PHIO Cellwatcher M microscope (PHIO scientific GmbH, Munich, Germany). The proliferation assays (2 independent assays) were analyzed using PHIOme Data Management and Analysis Platform software add-Ons “Proliferation” version 1.4.2. Two hypothesis tests were performed using Mann-Whitney U test and the Mood’s Median test. **B–E**: The figures show a cartographic representation of the change in cell coverage over time and thus representing the morphology. The individual cell areas are first extracted from microscopic images using image segmentation methods, and then their spread and morphology are plotted over time. Images are generated for each cell condition.

To shed light on the possible role of S1P in the context of chronic inflammation we selected a selective sphingosine-1-phosphate-1 receptor modulator AUY954, which experimentally reduces heart transplant rejection *in vivo.* AUY954 led to a significant higher anti-proliferative activity in human lung fibroblasts from COPD patients (DHLF-COPD) in comparison to normal lung fibroblasts (NHLF) (Fig. 5), suggesting that this pathway might be important in ECP and its mode of action. This is validated as well by cell life imaging using the PHIO Cellwatcher M by recording the drug effects 46 h (Fig. 6A) leading to a significant downregulation of proliferation only in the lung fibroblast from COPD patients. As drug concentration 7821 nM, the IC50 concentration of DHLF-COPD (Fig. 5) determined in the viability assay was used. In DHLF-COPD the Mann-Whitney U test showed a highly significant difference in distributions (U = 41246, *p* = 1.01 × 10^−5^) and Mood’s Median Test confirmed a significant difference in medians (χ^2^ = 15.82, *p* = 7 × 10^−5^) comparing control and treatment group while in NHLF no significant correlation could be detected (Fig. 6A).

In addition to that, AUY954 induces a morphological change in the DHLF-COPD to a more sprouted version (Fig. 6C, E) comparable to the NHLF while in NHLF no morphological change can be observed under AUY954 treatment (Fig. 6B, D).

## Discussion

Extracorporeal photopheresis represents an efficient and side-effect-free (or very low) treatment option for patients suffering from diseases such as cutaneous T-cell lymphoma and graft-versus-host diseases or to control organ transplant rejection.

Numerous beneficial clinical effects have been well documented but the underlying mode of action at the molecular level is not yet fully understood. The combination of mass spectrometry-based analysis of proteins and oxylipins in human plasma has the potential to detect characteristic biochemical processes in-vivo as previously demonstrated [37, 38]. Thus, here we performed such analyses using plasma from patients before and after ECP treatment on two consecutive days. Because these patients had different medical conditions, we expected a large variance in basal protein and lipid levels and wanted to identify the most robust treatment-specific effects. In addition, by doing so, we aimed to identify the common effects relevant for the different indications of ECP, as ECP is effective for all indications.

Although proteome analysis methodology has otherwise successfully identified disease- and lifestyle-associated proteome alterations [37, 38, 51, 52], in this case proteome profiling did not identify any such treatment-specific events.

Nevertheless, it is fair to conclude that ECP treatment had no detectable direct effect on plasma protein abundance values within the relatively short period of time during treatment. As patients receive ECP treatments over a longer time period (months) it cannot be excluded that ECP might in the long run induce specific proteome alterations. In addition, our cohort was rather small with different indications. Using PSVA we found “cellular response to lipid” and “regulation of plasminogen activation” to be the most significant regulated pathways, pointing out the involvement of the lipid metabolism and the possible involvement of the platelets in this context.

This is in contrast to the robust effects currently observed with fatty acids, oxylipins and the lipid mediator sphingosine-1-phosphate in ECP treatment. Here, a consistent lipid pattern was found to be significantly regulated and independently reproduced in two independent ECP treatment series and in all tested clinical indications such as CTCL, systemic sclerosis, chronic GvHD or acute or chronic rejection in heart transplants. Therefore, we highly suggest that these lipids are immediate regulators of ECP independent on the applied indication and might be the first mediators of the known anti-inflammatory effects of ECP treatment. The identified specific lipids excerpt anti-inflammatory activity and are outlined in the following.

Omega-3 polyunsaturated fatty acids (PUFA) including alpha-linolenic acid and EPA were found strongly upregulated upon ECP and are known to mediate anti-inflammatory effects *via* the inhibition of immune cell activation [53]. Although information on the immunomodulatory potential of stearidonic acid and DPA, which are also induced by ECP, is still limited, supplementation of stearidonic acid in an animal model reduced TNF-α expression upon lipopolysaccharide (LPS) stimulation in murine whole blood. In addition, alpha-linolenic acid, EPA and DPA levels in healthy human individuals were found negatively correlated with the inflammation score *via* a decrease of TNF-α and C-reactive protein (CRP) [54, 55].

In addition to omega-3 PUFAs, the omega-6 PUFA DGLA was also significantly upregulated upon ECP treatment. While it is generally assumed that a diet rich in omega-6 fatty acids promotes pro-inflammatory processes, recent studies also attribute anti-inflammatory effects to omega-6 PUFAs [56]. In freshly isolated human peripheral blood monocytes (PBMC), DGLA resulted in inhibition of TNF-α production upon LPS stimulation in-vitro, consistent with the suggested direct anti-inflammatory potential of omega-6 PUFAs [57]. Furthermore, attenuation of pro-inflammatory interferon-γ induced gene expression *via* dihomo-γ-linolenic acid (DGLA) was demonstrated in human and mouse macrophages [58]. In fact, omega-3 PUFA DHA and omega-6 PUFA AA were also found to be consistently upregulated by ECP treatment. Due to the limited sample size, significance thresholds were not reached.

The linoleic acid oxidation product 13-OxoODE, formed *via* the 15-lipoxygenase (LOX) pathway, has been described as potent anti-inflammatory endogenous peroxisome proliferation-activated receptor gamma (PPAR-γ) agonist in macrophages or human colonic epithelial cells [59, 60]. Remarkably, 13-OxoODE was among the significantly upregulated anti-inflammatory molecular pattern in the plasma of patients suffering from long COVID-19 syndrome compared to fully recovered COVID-19 patients [37].

Saturated fatty acids such as stearic acid (C18:0) are known contributors to the development of cardiovascular diseases, and stearic acid in particular has been described to mediate pro-inflammatory effects and cell growth inhibition in human aortic endothelial cells *via* upregulation of intercellular adhesion molecule-1 (ICAM-1), which regulates leukocyte recruitment to the site of inflammation [61], and the induction of nuclear factor-kappa B (NF-κB) signaling [62]. Therefore, the significant downregulation of stearic acid upon ECP treatment may also represent an anti-inflammatory effect *via* potentially reduced leukocyte recruitment.

In addition, upregulation of 12-HETE, 12-HEPE and 14-HDoHE, which are formed *via* the 12-LOX pathway of AA, EPA and DHA, respectively, was also found in ECP [63]. The so-called “platelet-type” 12-LOX is mainly expressed in platelets [64] and we have previously observed the release of 12-LOX derived oxylipins *via* activation of isolated platelets in-vitro This observation may suggest that some metabolic activation of platelets may occur with ECP treatment. ECP is known to modulate the immune system, has anti-inflammatory properties in the different indications and helps to reduce the dosages of the treatment with glucocorticoids and immunosuppressive drugs. Consequently, rate of opportunistic infections can be lowered through ECP.

Sphingosine-1-phosphate (S1P) is a potent signaling lipid mainly derived from red blood cells, platelets, macrophages, mast cells and endothelial cells [65] and is a strong promotor of inflammasome activation [66, 67]. S1P can specifically activate five different G-protein coupled receptors (GPCR) regulating various physiologic processes including the promotion of inflammation [68]. The beneficial effects of extracorporeal photopheresis may thus also be attributed to the downregulation of S1P observed upon ECP treatment.

As a next step, we used the S1P receptor 1 modulator AUY954 to selectively decrease S1P1 levels and to analyze the effects of S1P receptor signaling in normal human lung fibroblasts and lung fibroblasts from COPD patients, in a chronic inflammatory setting. ECP is used in the context of lung transplantation in COPD patients and is known to have anti-fibrotic and anti-inflammatory effects. In addition, the S1P modulators are recently evaluated in various indications such as autoimmune diseases, dermatomyositis, Crohn’s disease, ulcerative colitis, polymyositis, GvHD and transplant rejection [69], which are also indications for ECP suggesting that similar effects are underlying. This is also underlined by the mechanisms of S1P modulators, in which absence of S1P blocks lymphocyte proliferation and reduces T and B cell counts in the blood, leading to immune suppression. The selective S1P agonist AUY954 reduced circulating lymphocytes and prolonged cardiac allograft survival in vivo [70] and inhibited inflammatory demyelination and immune cell infiltration in an animal model of experimental autoimmune neuritis [71]. Here we demonstrate that AUY954 selectively inhibits proliferation in fibroblasts from COPD patients compared to normal lung fibroblasts, suggesting that the anti-fibrotic and anti-inflammatory effects of ECP may be partially mediated by S1P and that S1P downregulation might be one of the key mechanisms of ECP. The cell morphology of DHLF-COPD appears to be converted to a normal lung fibroblast phenotype. In addition, we provide evidence that the combination with lipid modulators may be synergistic, as they have immunosuppressive effects and are used in comparable indications.

### Conclusions and expert recommendation in the framework of PPPM

While the ECP-induced lipid mediators have well known anti-inflammatory properties and could therefore account for the clinical observations among all different clinical indications, the molecular mechanism resulting in the increase of these lipids is not yet clear. Future studies will help identify the potential contribution of platelets, erythrocytes, leukocytes, or other blood components, will elucidate long-term effects and can shed light on the question if further inflammatory diseases might be indications for ECP. In addition, these effects might also account for PUVA therapy used i.e. in inflammatory skin diseases, since ECP is a therapeutic concept derived from PUVA therapy. It is evident that identifying ECP-induced changes in lipid mediators and fatty acid composition will contribute to a better understanding of the mode of action of this important intervention strategy.

The influence of lipids on the mode of action of ECP is a complex and cutting-edge field of research and medicine. Lipidomics requires specific expertise in mass spectrometry and specific bioinformatics analysis and interpretation, and the management of different internal medicine and dermatological diagnoses requires interdisciplinary expertise. The in-depth study of the effects of ECP had made great innovations in the following three cornerstones of PPPM:

1. Predictive approach. Here we demonstrate a significant upregulation of anti-inflammatory lipids under ECP. In a next step, we will evaluate these lipids in a long-term study at baseline level and over time and correlated with response. As ECP patients need peripheral access from a cubital vein, blood sampling would not be an additional intervention. Due to the importance of lipids in anti-inflammatory processes, we hypothesize, that these candidates might serve as predictive and prognostic biomarkers.
2. Targeted prevention. Interestingly, ECP is used or evaluated at all three levels of targeted prevention. In our setting, we used the most frequently applied and clinically established level of prevention of ECP, the tertiary level. By using different indications of ECP we were able to demonstrate that ECP influences the lipids on the tertiary level of prevention irrespective of the indication. In addition, ECP is evaluated in a prophylactic manner for transplant patients to eventually prevent acute or chronic organ rejection. Here we suggest that the depicted anti-inflammatory lipids might be important players in all three settings.
3. Personalization of medical services. Here we present different anti-inflammatory lipids consistently regulated on two independent days under ECP. The individual lipid status might give information if ECP therapy is recommended and if new combinatorial therapeutic concepts with lipid modulators might be beneficial.

To the best of our knowledge, this is the first study presenting well-defined molecular changes at the lipid level induced by ECP, which may explain important aspects of its mode of action. In conclusion, the presented data suggest that the lipid status offers many opportunities to support personalized medicine in the primary, secondary and tertiary care of prevention, ranging from the identification of reliable predictive biomarker patterns, to the assessment of lipid status for novel treatment options, to the monitoring of therapeutic efficacy. Further studies using additional time points, larger cohorts of responders and non-responders, and evaluating different combinatorial time points are warranted.

## Electronic supplementary material

Below is the link to the electronic supplementary material.

Supplementary Material 1

Supplementary Material 2

Supplementary Material 3

Supplementary Material 4

Supplementary Material 5

Supplementary Material 6

Supplementary Material 7

Supplementary Material 8

## Data Availability

No datasets were generated or analysed during the current study.
