# Supplementary material for: Extracorporeal photopheresis induces the release of anti-inflammatory fatty acids and oxylipins and suppresses pro-inflammatory sphingosine-1-phosphate
Source: Inflamm Res. 2025 Feb 13;74(1):40. doi: 10.1007/s00011-025-02007-6 (PMC11825557; doi:10.1007/s00011-025-02007-6)
Supplement: Supplementary file 1 — Supplementary Material 1 [file 11_2025_2007_MOESM1_ESM.docx]

| **A** | **B** |
| --- | --- |
|  |  |
| **Supplementary Figure 1:** Volcano plots of **A**: day 2 and day 1 before ECP and **B**: day 2 and day 1 after ECP, log_2_FC on the x-axis, -log_10_adj.Pval on the y-axis. | |
