# Supplementary material for: Extracorporeal photopheresis induces the release of anti-inflammatory fatty acids and oxylipins and suppresses pro-inflammatory sphingosine-1-phosphate
Source: Inflamm Res. 2025 Feb 13;74(1):40. doi: 10.1007/s00011-025-02007-6 (PMC11825557; doi:10.1007/s00011-025-02007-6)
Supplement: Supplementary file 2 — Supplementary Material 2 [file 11_2025_2007_MOESM2_ESM.docx]

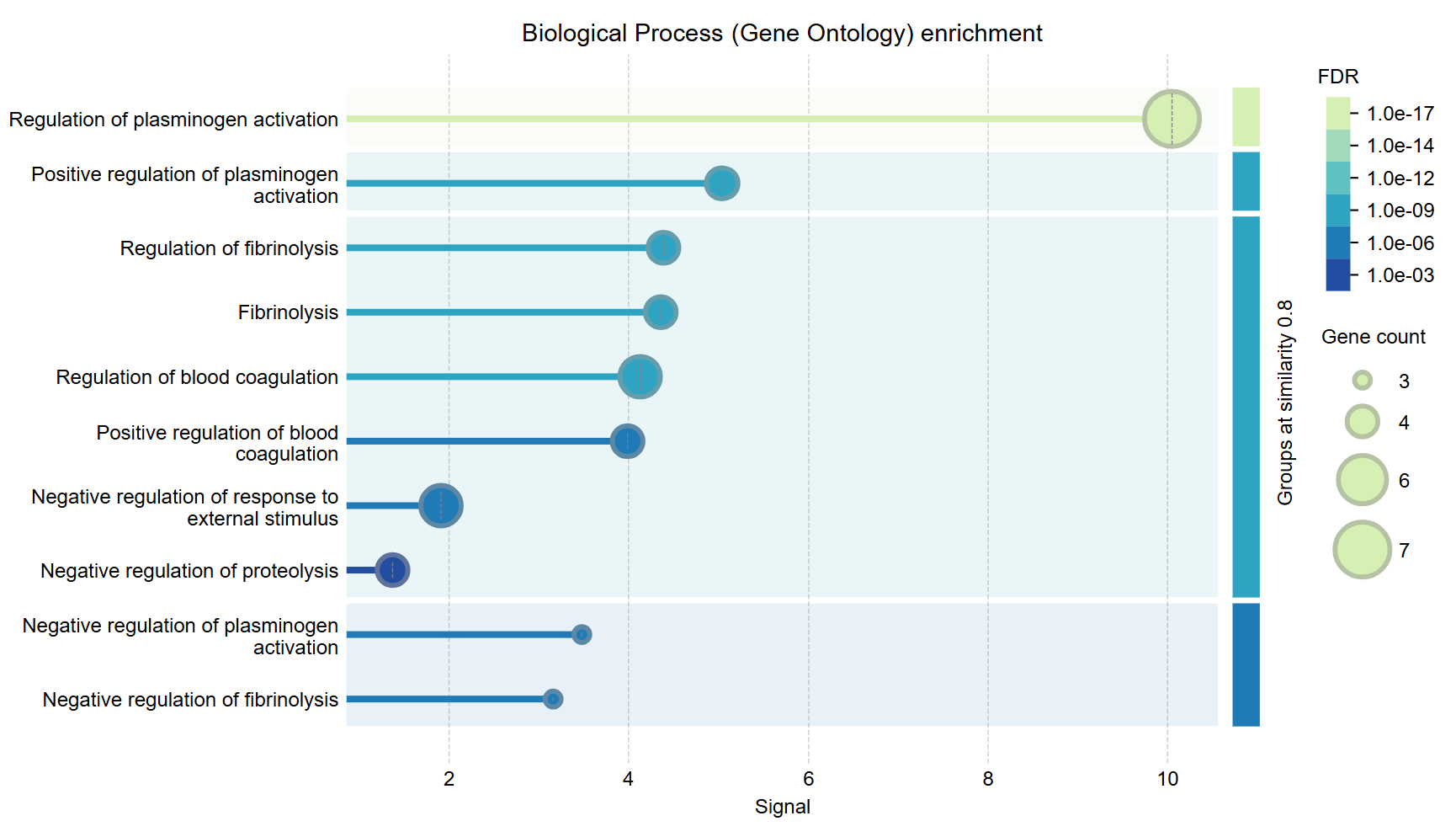

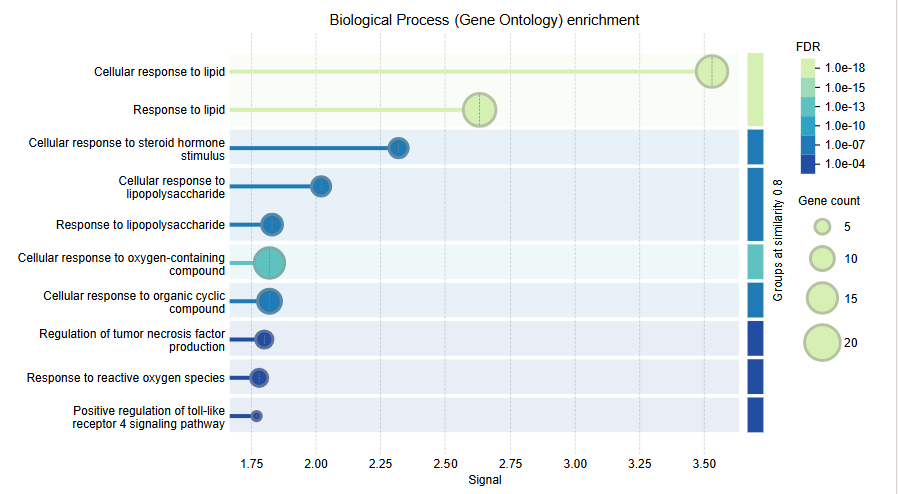

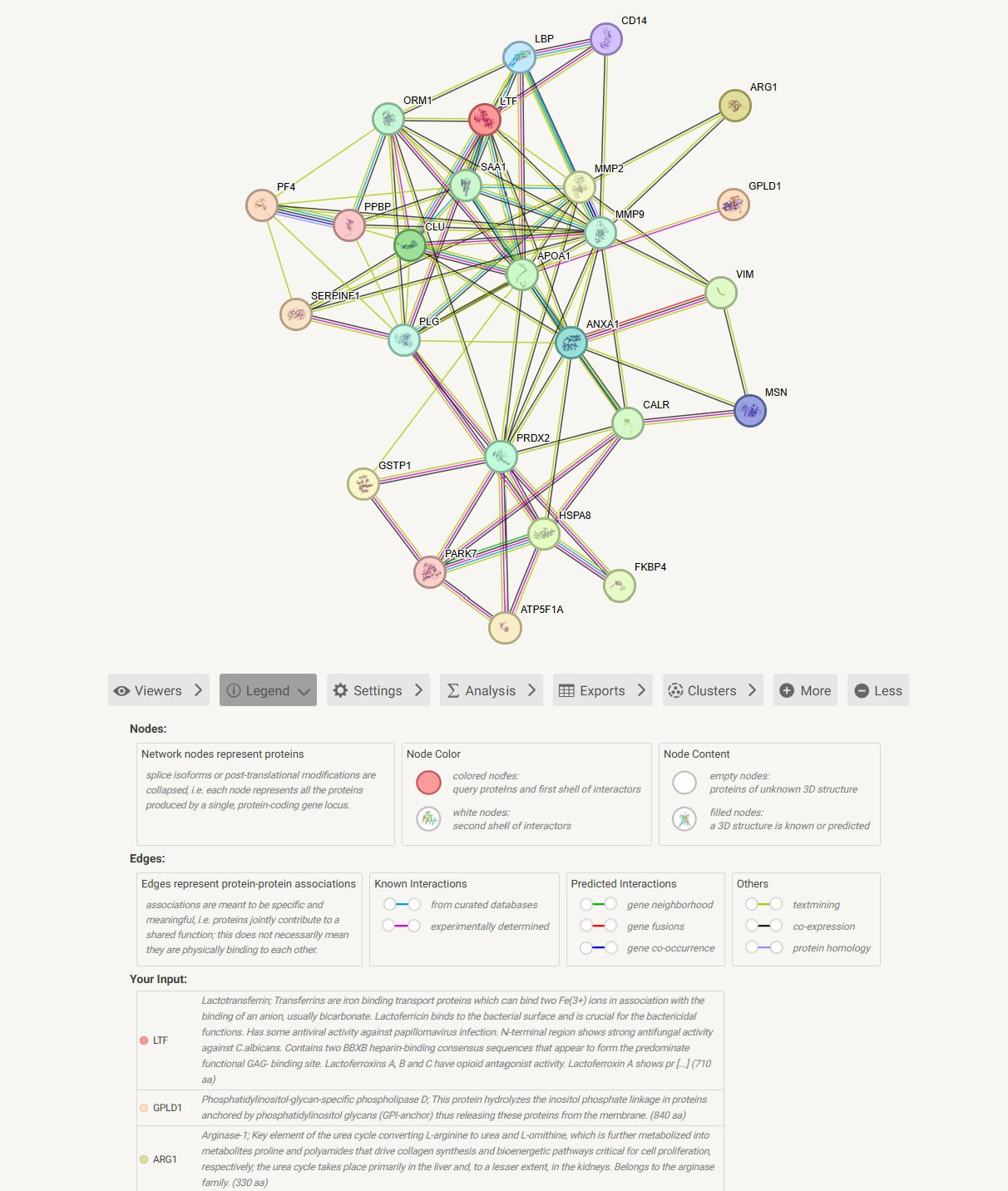

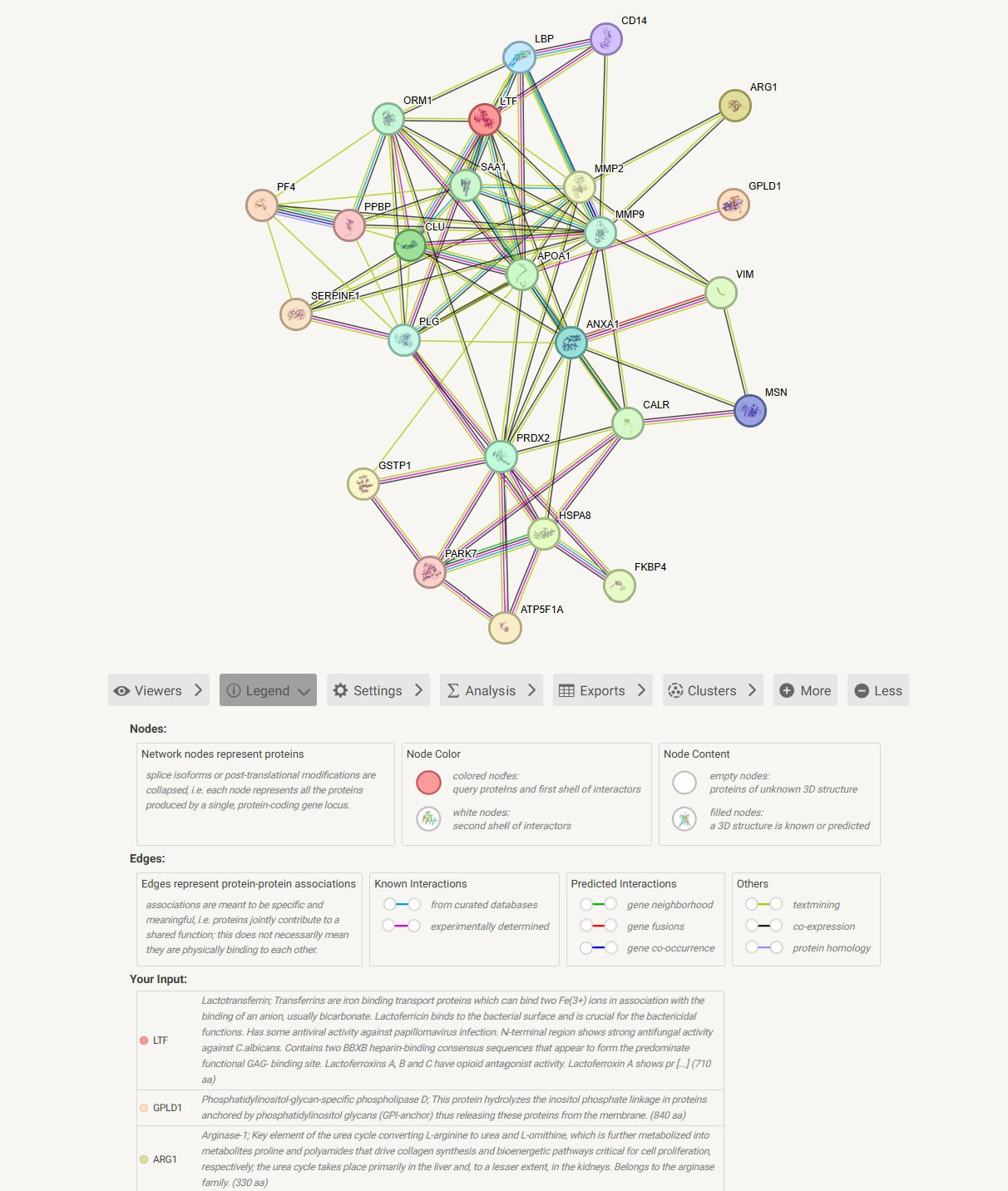

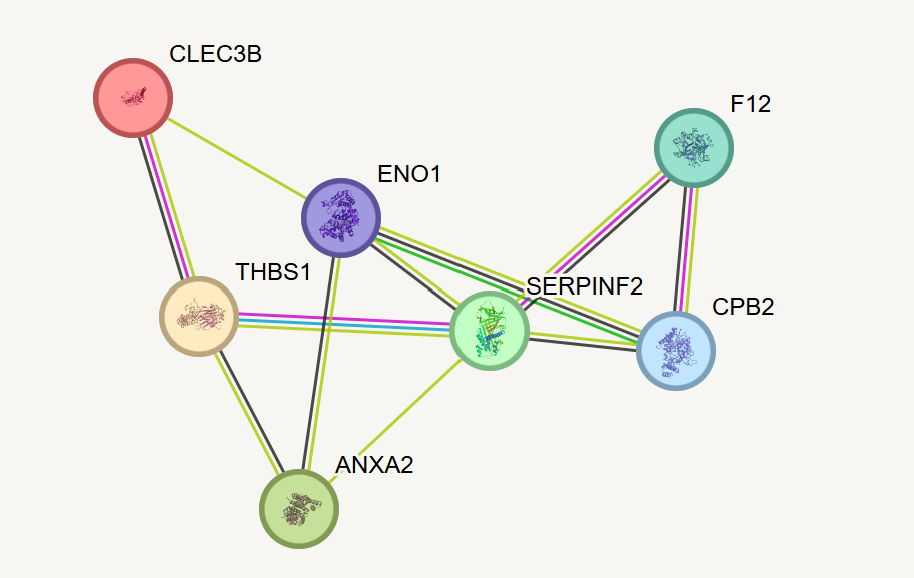


**Supplementary Figure 2:** String analysis of proteins involved in the most significant regulated pathways A: day 1 “cellular response to lipid” and B: day 2 “regulation of plasminogen activation”

A: day 1

B: day 2
